# Supplementary material for: Content-rich biological network constructed by mining PubMed abstracts
Source: BMC Bioinformatics. 2004 Oct 8;5:147. doi: 10.1186/1471-2105-5-147 (PMC528731; doi:10.1186/1471-2105-5-147)
Supplement: Additional File 5 — The original Chilibot query results of the term "long-term potentiation (LTP)" and 22 other terms, limiting the latest references analyzed to the years 1990, 1995, 2000, and 2004. [file 1471-2105-5-147-S5.bz2 › chilibotAdditionalFile5/ltp2000/html/PKC_ACTIN.html]

 


 **PKC** and **ACTIN** 
  
Found 355 abstracts in PubMed,  **30 abstracts were retrieved and analyzed**.  


---

 Search Google  |
 PDF files only 
|  EDU domain only 

---

**Interactive relationship** (e.g. stimulation, inhibition, etc)

- GAP43, myristoylated alanine rich C kinase substrate MARCKS, and CAP23 GMC are locally abundant, plasmalemma associated  **PKC**  substrates that affect  **actin**  cytoskeleton.  Ref: 10871285 J Cell Biol, 2000
- Myristoylated alanine rich C kinase substrate MARCKS, as a specific protein kinase C  **PKC**  substrate, mediates  **PKC**  signaling through its phosphorylation and subsequent modification of its association with filamentous  **actin**  F  **actin**  and calmodulin CaM.  Ref: 11029309 Am J Physiol Cell Physiol, 2000
- Myristoylated alanine rich C kinase substrate MARCKS, a protein specifically phosphorylated by protein kinase C  **PKC** , crosslinks  **actin**  filaments and anchors the  **actin**  network to the inner leaflet of the PM.  Ref: 10859236 Biol Reprod, 2000
- Myristoylated alanine rich C kinase substrate MARCKS, as a specific  **PKC**  substrate, appears to mediate  **PKC**  signaling by  **PKC**  dependent phosphorylation of MARCKS and subsequent modification of the association of MARCKS with filamentous  **actin**  and calmodulin CaM.  Ref: 10972986 J Cell Biochem, 2000
- Adducin is a ubiquitously expressed membrane skeletal protein localized at spectrin  **actin**  junctions that binds calmodulin and is an in vivo substrate for protein kinase C  **PKC**  and Rho associated kinase.  Ref: 10950304 Cell Mol Life Sci, 2000
- When chromaffin cells are stimulated through nicotinic receptors, cortical F  **actin**  disassembly is mainly through the intervention of pathway A, since in the presence of  **PKC**  inhibitors, F  **actin**  disassembly in response to cholinergic stimulation is only blocked by 20%.  Ref: 10865122 Biochimie, 2000
- Calponin, an F  **actin**  associated protein implicated in the regulation of smooth muscle contraction, is known to be phosphorylated in vitro by protein kinase C  **PKC**  and calcium calmodulin dependent protein kinase II CaM kinase II.  Ref: 10873572 Biochem Biophys Res Commun, 2000
- Calponin inhibits  **actin**  myosin interaction in a manner that is prevented by protein kinase C  **PKC**  catalysed phosphorylation of serine 175.  Ref: 10947974 Biochem J, 2000
- Unlike store mediated calcium entry, jasplakinolide, which reorganises  **actin**  filaments into a tight cortical layer adjacent to the plasma membrane, did not inhibit divalent cation influx evoked by thrombin when applied after calcium store depletion, or by activators of  **PKC** .  Ref: 11080259 J Physiol, 2000
- Pathway B is fully activated by phorbol esters and in this case  **PKC**  blockers inhibit by 100% the disruption of cortical F  **actin** .  Ref: 10865122 Biochimie, 2000
- Together, these results suggest that upon activation of neural adhesion molecules, the action of  **PKC**  and p190 RhoGAP leads to a modulation of Rho GTPase activity to direct several  **actin**  dependent morphogenetic processes required for normal neural development.  Ref: 11044403 Development, 2000

**Parallel relationship** (e.g. studied together, co-existance, homology, etc.)

- Upon either direct activation of  **PKC**  or in response to integrin engagement, p190 RhoGAP is rapidly translocated to regions of membrane ruffling, where it colocalizes with polymerized  **actin** .  Ref: 11044403 Development, 2000
- In the present study, we examined possible involvement of protein kinase C  **PKC**  and extracellular signal regulated protein kinase Erk 1 in chondrogenesis of mesenchymal cells induced by disruption of  **actin**  cytoskeleton.  Ref: 10873653 Biochem Biophys Res Commun, 2000
- Double labeling experiments with CSK demonstrate that  **PKC**   **actin**  co localizes with punctate alpha rich particles above the nucleus, granules of epsilon throughout the cytoplasm, and with theta in irregular shaped aggregates associated with the nucleus.  Ref: 11118478 J Histochem Cytochem, 2001
- Upon activation of  **PKC**  in vivo, sm22 dissociates from the  **actin**  cytoskeleton and is distributed diffusely in the cytoplasm.  Ref: 10939458 Electrophoresis, 2000
- These data indicate that retinoid treatment causes inactivation of  **PKC**  alpha, allowing MARCKS to relocalize to the membrane, where it can cross link  **actin**  filaments.  Ref: 11115359 Exp Mol Pathol, 2000
